# Supplementary material for: Conserved microRNA targeting reveals preexisting gene dosage sensitivities that shaped amniote sex chromosome evolution
Source: Genome Res. 2018 Apr;28(4):474–83. doi: 10.1101/gr.230433.117 (PMC5880238; doi:10.1101/gr.230433.117)
Supplement: Supplemental Material [file supp_28_4_474__index.html]

Conserved microRNA targeting reveals preexisting gene dosage sensitivities that shaped amniote sex chromosome evolution — Supplemental Material 

# Conserved microRNA targeting reveals preexisting gene dosage sensitivities that shaped amniote sex chromosome evolution

## Supplemental Material

- Supplemental\_Fig\_S1.pdf
- Supplemental\_Fig\_S2.pdf
- Supplemental\_Fig\_S3.pdf
- Supplemental\_Fig\_S4.pdf
- Supplemental\_Fig\_S5.pdf
- Supplemental\_Fig\_S6.pdf
- Supplemental\_Fig\_S7.pdf
- Supplemental\_Fig\_S8.pdf
- Supplemental\_Fig\_S9.pdf
- Supplemental\_Fig\_S10.pdf
- Supplemental\_Fig\_S11.pdf
- Supplemental\_Fig\_S12.pdf
- Supplemental\_Fig\_S13.pdf
- Supplemental\_Fig\_S14.pdf
- Supplemental\_Fig\_S15.pdf
- Supplemental\_Fig\_S16.pdf
- Supplemental\_Fig\_S17.pdf
- Supplemental\_Fig\_S18.pdf
- Supplemental\_Fig\_S19.pdf
- Supplemental\_Table\_S1.xlsx
- Supplemental\_Table\_S6.xlsx
- Supplemental\_Code.zip
- Supplemental\_Methods.docx
- Supplemental\_Table\_S2.xlsx
- Supplemental\_Table\_S3.xlsx
- Supplemental\_Table\_S4.xlsx
- Supplemental\_Table\_S5.txt
