# Supplementary material for: Conserved microRNA targeting reveals preexisting gene dosage sensitivities that shaped amniote sex chromosome evolution
Source: Genome Res. 2018 Apr;28(4):474–83. doi: 10.1101/gr.230433.117 (PMC5880238; doi:10.1101/gr.230433.117)
Supplement: Supplemental Material [file supp_gr.230433.117_Supplemental_Fig_S1.pdf]

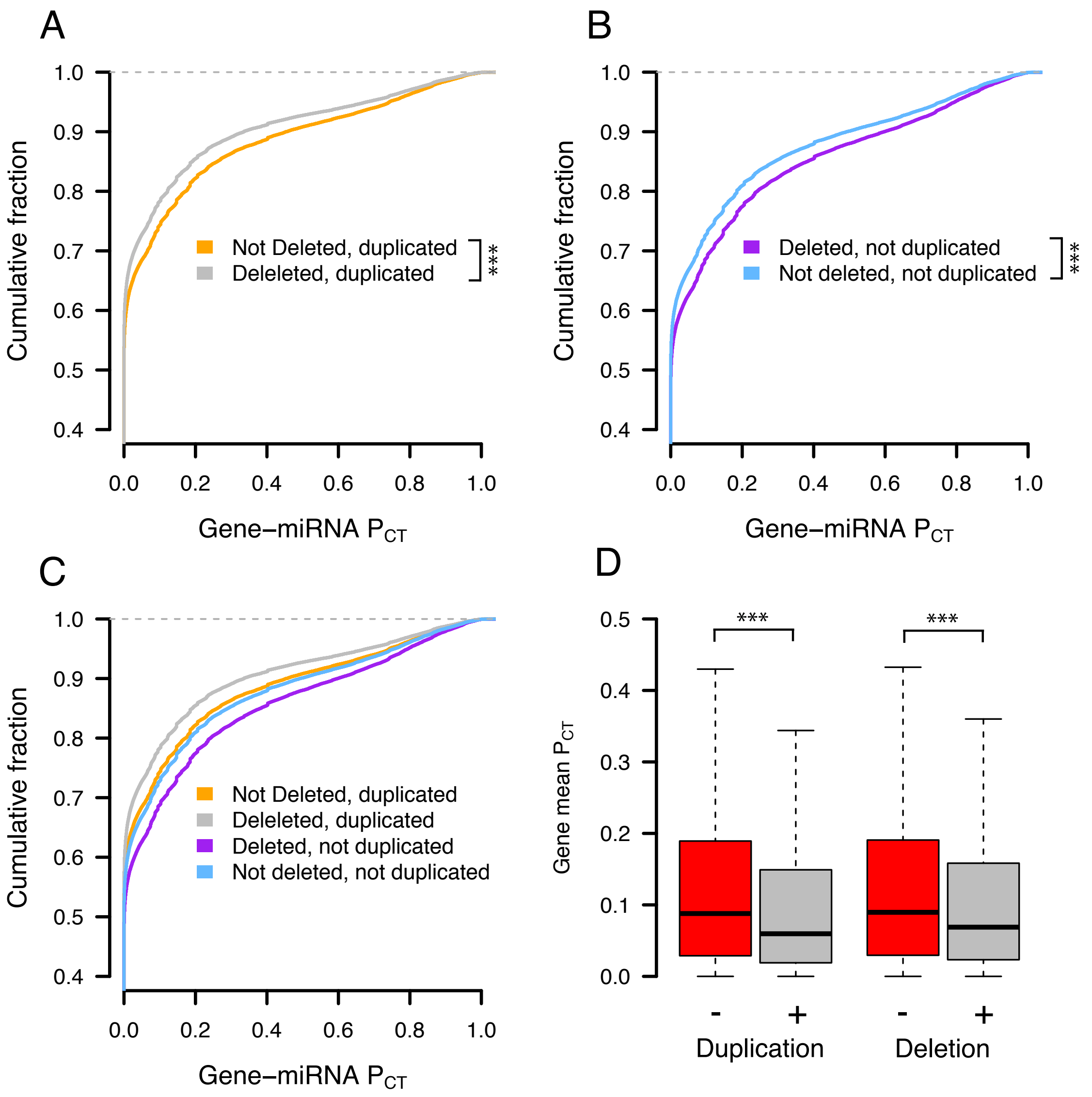

Supplemental Figure S1: Effect of deletion status on autosomal P_{CT} scores. Probabilities of conserved targeting (P_{CT}) of all gene-miRNA interactions involving non-deleted and deleted genes, further stratified as (A) duplicated (grey, n = 69,339 interactions from 4,118 genes; orange, n = 51,514 interactions from 2,916 genes) or (B) not duplicated (purple, n = 72,826 interactions from 3,510 genes; blue, n = 80,290 interactions from 3,976 genes). *** $p < 0.001$, two-sided Kolmogorov-Smirnov test. (C) P_{CT} scores for all gene sets in (A) and (B) superimposed on one plot. (D) Mean gene-level P_{CT} scores when aggregating sets of duplicated/not duplicated (left) or deleted/not deleted (right) genes. *** $p < 0.0001$, two-sided Wilcoxon rank-sum test.
