# Supplementary material for: Conserved microRNA targeting reveals preexisting gene dosage sensitivities that shaped amniote sex chromosome evolution
Source: Genome Res. 2018 Apr;28(4):474–83. doi: 10.1101/gr.230433.117 (PMC5880238; doi:10.1101/gr.230433.117)
Supplement: Supplemental Material [file supp_gr.230433.117_Supplemental_Fig_S2.pdf]

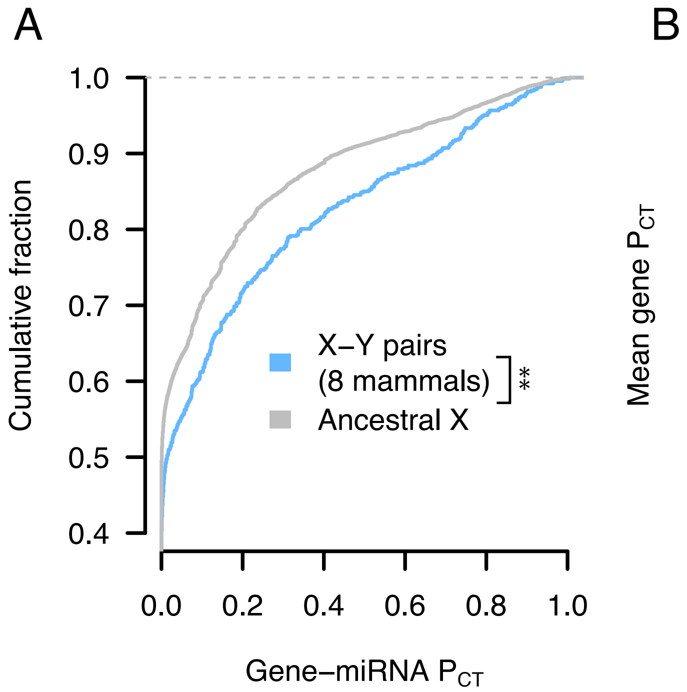

Supplemental Figure S2: P_{CT} scores of X-Y pairs across 8 mammals. (A) P_{CT} score distributions of all gene-miRNA interactions involving X-Y pairs across eight sequenced mammalian Y Chromosomes (n = 647 interactionsfrom 32 genes) and other ancestral X genes (n = 8,831 interactions from 457 genes). ** $p < 0.01$, two-sided Kolmogorov-Smirnov test. (B) Gene-level mean P_{CT} scores. * $p < 0.05$, two-sided Wilcoxon rank-sum test.
