# Supplementary material for: Conserved microRNA targeting reveals preexisting gene dosage sensitivities that shaped amniote sex chromosome evolution
Source: Genome Res. 2018 Apr;28(4):474–83. doi: 10.1101/gr.230433.117 (PMC5880238; doi:10.1101/gr.230433.117)
Supplement: Supplemental Material [file supp_gr.230433.117_Supplemental_Fig_S3.pdf]

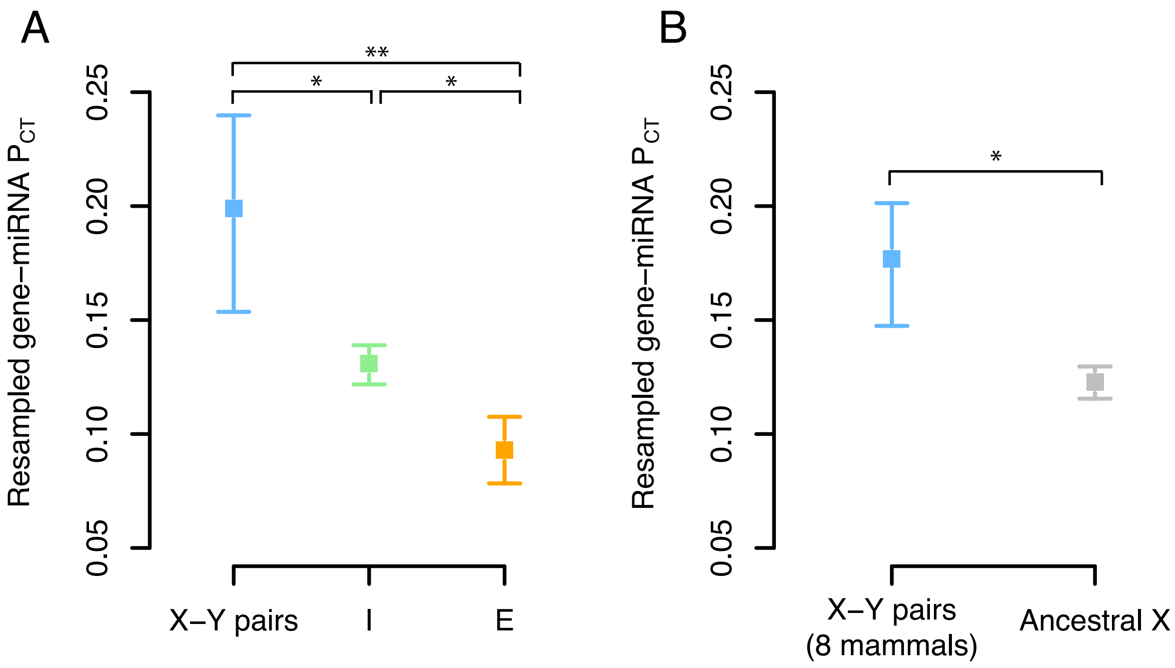

Supplemental Figure S3: Resampled mean P_{CT} scores of X-linked genes. (A) Resampled gene-miRNA P_{CT} scores for human X-Y pairs (n = 15 genes), X-inactivated genes (n = 329 genes) and X escape genes (n = 56 genes). (B) Resampled gene-miRNA P_{CT} scores for X-Y pairs across eight mammals (n = 32 genes) and genes with no Y homolog in any of eight mammals (n = 457 genes). Points and error bars represent the median and 95% confidence intervals from 1,000 gene samplings with replacement. * $p < 0.05$, ** $p < 0.01$, empirical p-value computed as the fraction of random non-overlapping gene sets with a median difference in P_{CT} score at least as large as the true difference.
