# Supplementary material for: Conserved microRNA targeting reveals preexisting gene dosage sensitivities that shaped amniote sex chromosome evolution
Source: Genome Res. 2018 Apr;28(4):474–83. doi: 10.1101/gr.230433.117 (PMC5880238; doi:10.1101/gr.230433.117)
Supplement: Supplemental Material [file supp_gr.230433.117_Supplemental_Fig_S4.pdf]

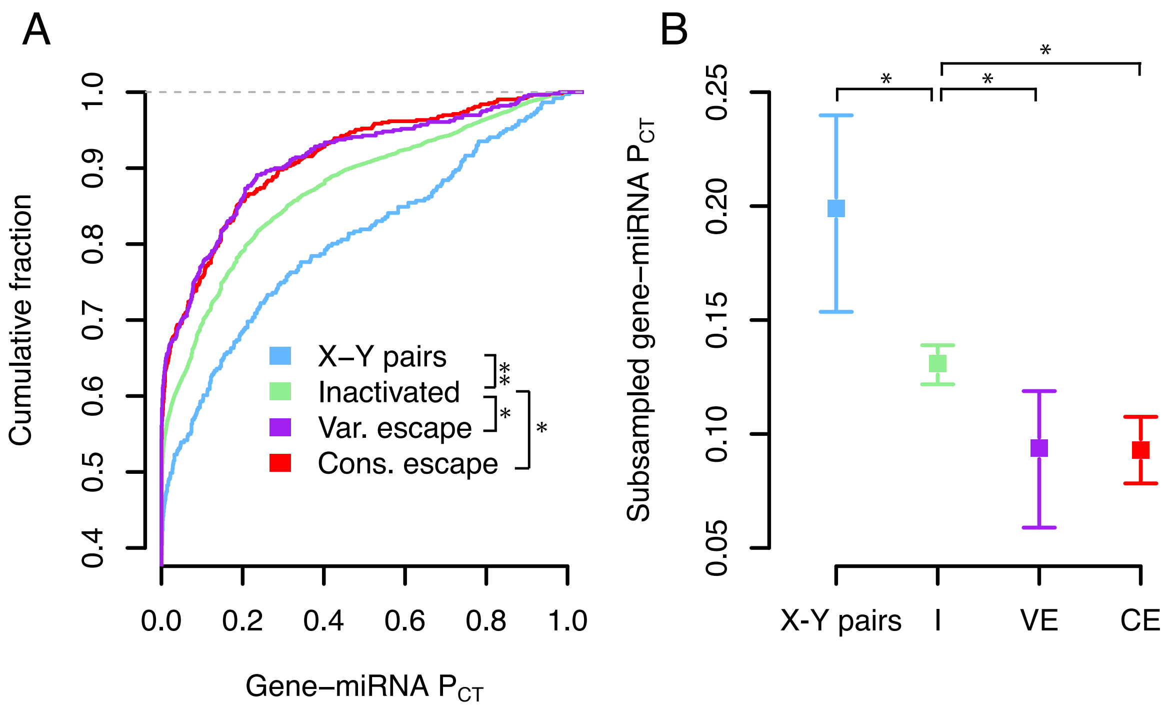

Supplemental Figure S4: P_{CT} score comparisons with consistent and variable escape genes separated. (A) P_{CT} score distributions of all gene-miRNA interactions involving X-Y pairs (n = 371 interactions from 16 genes), X-inactivated genes (n = 6743 interactions from 329 genes), consistent escape genes (n = 567 interactions from 30 genes), or variable escape genes (n = 470 interactions from 26 genes) as defined by Balaton et al (Balaton et al., 2015). * $p < 0.05$, ** $p < 0.01$, two-sided Kolmogorov-Smirnov test. (B) Resampled gene-miRNA P_{CT} scores of gene classes from (A). Points and error bars represent the median and 95% confidence intervals from 1,000 gene samplings with replacement. * $p < 0.05$, empirical p-value computed as the fraction of random non-overlapping gene sets with a median difference in P_{CT} score at least as large as the true difference.
