# Supplementary material for: Conserved microRNA targeting reveals preexisting gene dosage sensitivities that shaped amniote sex chromosome evolution
Source: Genome Res. 2018 Apr;28(4):474–83. doi: 10.1101/gr.230433.117 (PMC5880238; doi:10.1101/gr.230433.117)
Supplement: Supplemental Material [file supp_gr.230433.117_Supplemental_Fig_S5.pdf]

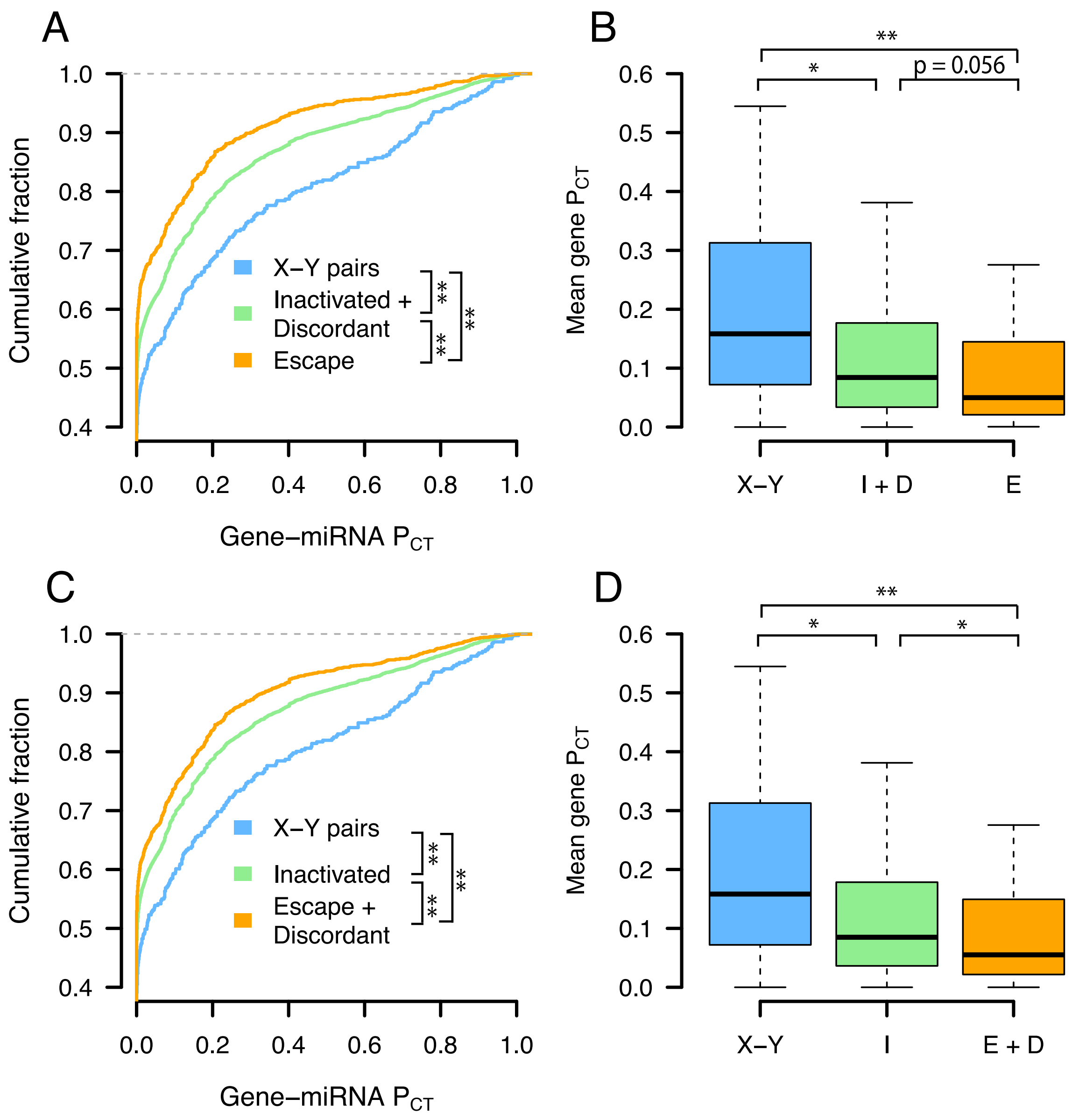

Supplemental Figure S5: P_{CT} score comparisons with discordant genes included as X-inactivated or escape. P_{CT} score distributions of all gene-miRNA interactions (A,C) or mean gene-level P_{CT} score (B,D) of classes of X-linked genes with genes with a discordant XCI call (n = 721 interactions from 40 genes) included as X-inactivated (A,B) or X escape (C,D). Numbers of gene-miRNA interactions and genes as in Figure 1, but with the addition of discordant gene numbers/interactions to X-inactivated genes (A,B) or X escape genes (C,D). * $p < 0.05$, ** $p < 0.01$, two-sided Kolmogorov-Smirnov (A,C) or Wilcoxon rank-sum (B,D) test.
