# Supplementary material for: Conserved microRNA targeting reveals preexisting gene dosage sensitivities that shaped amniote sex chromosome evolution
Source: Genome Res. 2018 Apr;28(4):474–83. doi: 10.1101/gr.230433.117 (PMC5880238; doi:10.1101/gr.230433.117)
Supplement: Supplemental Material [file supp_gr.230433.117_Supplemental_Fig_S6.pdf]

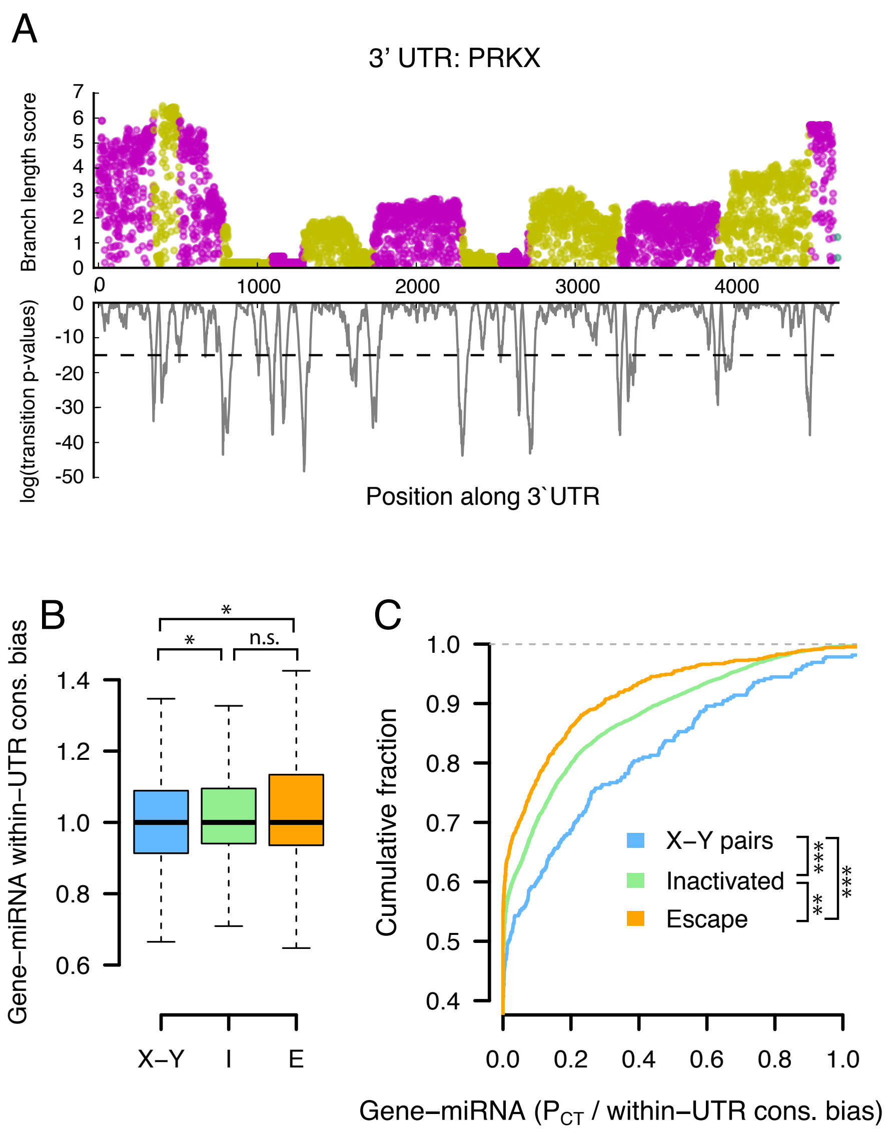

Supplemental Figure S6: Variation in within-UTR conservation does not account for observed differences in P_{CT} score among classes of X-linked genes. (A) Example of step-detection to segment 3` UTRs. Top, base-wise branch length scores; bottom, probabilities of transition to a new section. Dashed line indicates p-value cutoff used to delineate a new section (plotted as alternating magenta/yellow points). (B) Boxplots of within-UTR conservation bias (see Methods) for all gene-miRNA interactions involving classes of X-linked genes. (C) Comparisons of P_{CT} scores normalized by within-UTR bias. **, $p < 0.01$, *** $p < 0.001$, two-sided Kolmogorov-Smirnov test.
