# Supplementary material for: Conserved microRNA targeting reveals preexisting gene dosage sensitivities that shaped amniote sex chromosome evolution
Source: Genome Res. 2018 Apr;28(4):474–83. doi: 10.1101/gr.230433.117 (PMC5880238; doi:10.1101/gr.230433.117)
Supplement: Supplemental Material [file supp_gr.230433.117_Supplemental_Fig_S7.pdf]

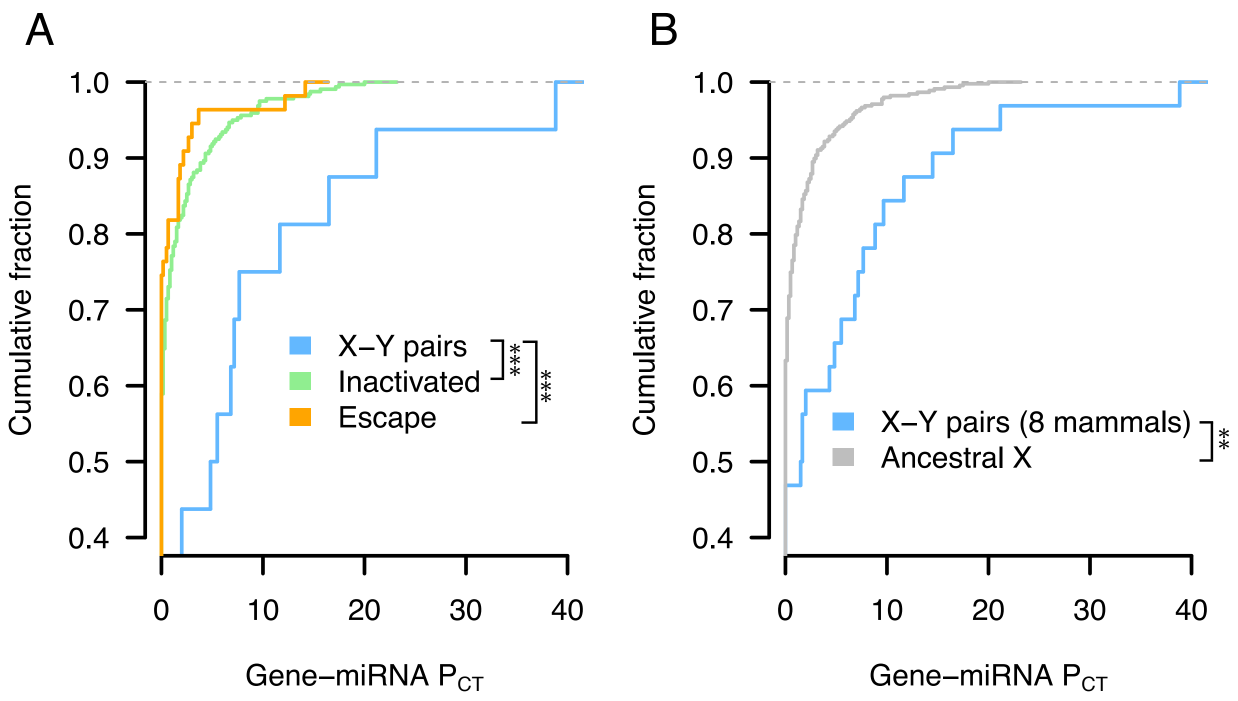


**Supplemental Figure S7: Background human-chicken 3' UTR conservation among classes of X-linked genes.** Mean number of human-chicken conserved sites found using shuffled miRNA seed sequences for (A) human X-Y pairs (n = 15 genes), X-inactivated genes (n = 329 genes) and X escape genes (n = 56 genes), and (B) X-Y pairs across eight mammals (n = 32 genes) and genes with no Y homolog in any of eight mammals (n = 457 genes). *** $p < 0.001$, two-sided Wilcoxon rank-sum test.
