# Supplementary material for: Conserved microRNA targeting reveals preexisting gene dosage sensitivities that shaped amniote sex chromosome evolution
Source: Genome Res. 2018 Apr;28(4):474–83. doi: 10.1101/gr.230433.117 (PMC5880238; doi:10.1101/gr.230433.117)
Supplement: Supplemental Material [file supp_gr.230433.117_Supplemental_Fig_S8.pdf]

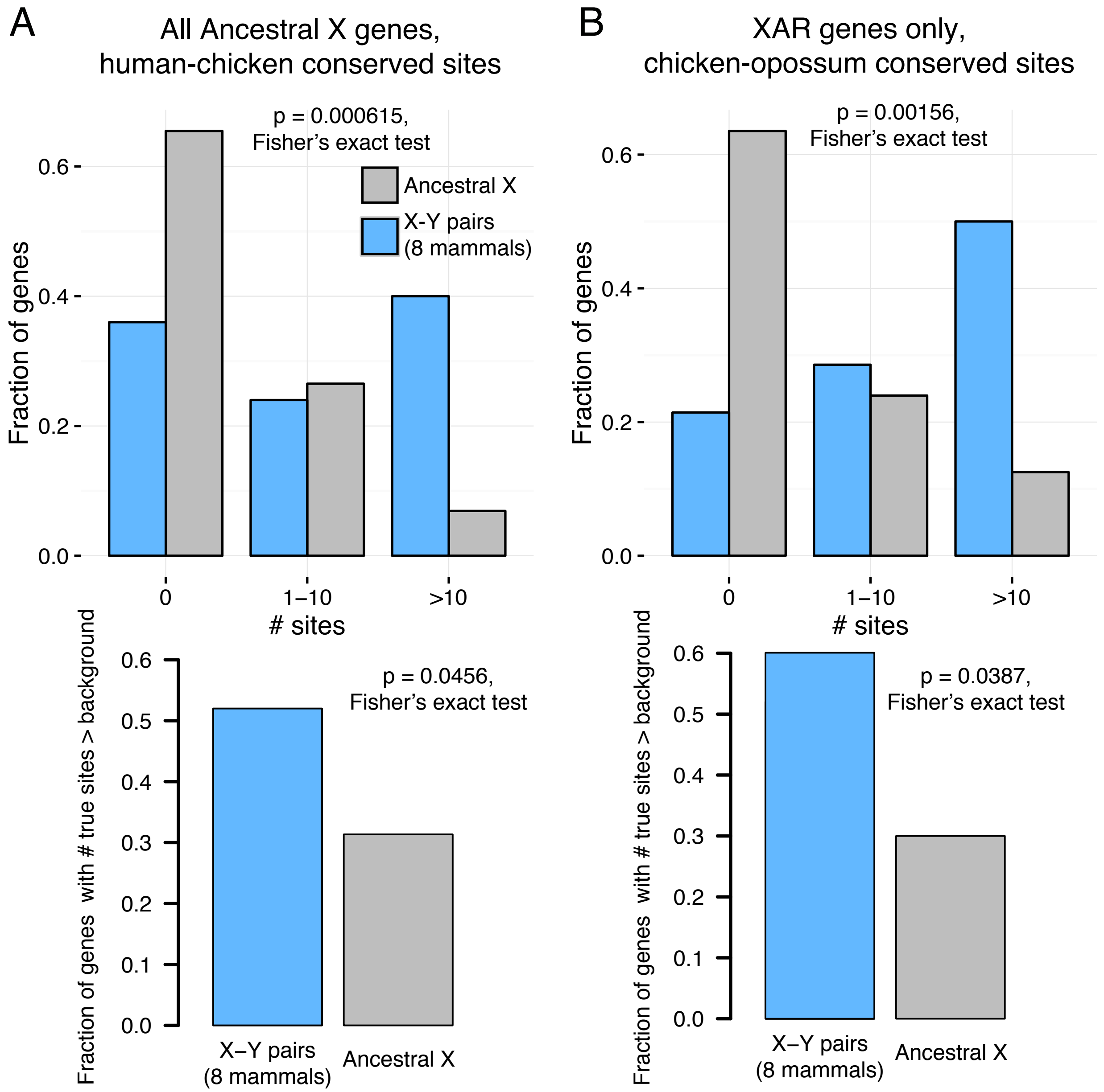

Supplemental Figure S8: Ancestral miRNA targeting of X-Y pairs across 8 mammals. (A) Distributions of sites conserved between 3` UTRs of human and chicken orthologs (top) or comparisons to background expectation (bottom, see Methods) for X-Y pairs across 8 mammals (n = 25) and other ancestral X genes (n = 351). (D) Statistics as in (C), but using sites conserved between chicken and opossum 3` UTRs only for genes in the XAR; X-Y pairs across 8 mammals (n = 15), other ancestral X genes (n = 102).
