# Supplementary material for: Conserved microRNA targeting reveals preexisting gene dosage sensitivities that shaped amniote sex chromosome evolution
Source: Genome Res. 2018 Apr;28(4):474–83. doi: 10.1101/gr.230433.117 (PMC5880238; doi:10.1101/gr.230433.117)
Supplement: Supplemental Material [file supp_gr.230433.117_Supplemental_Fig_S9.pdf]

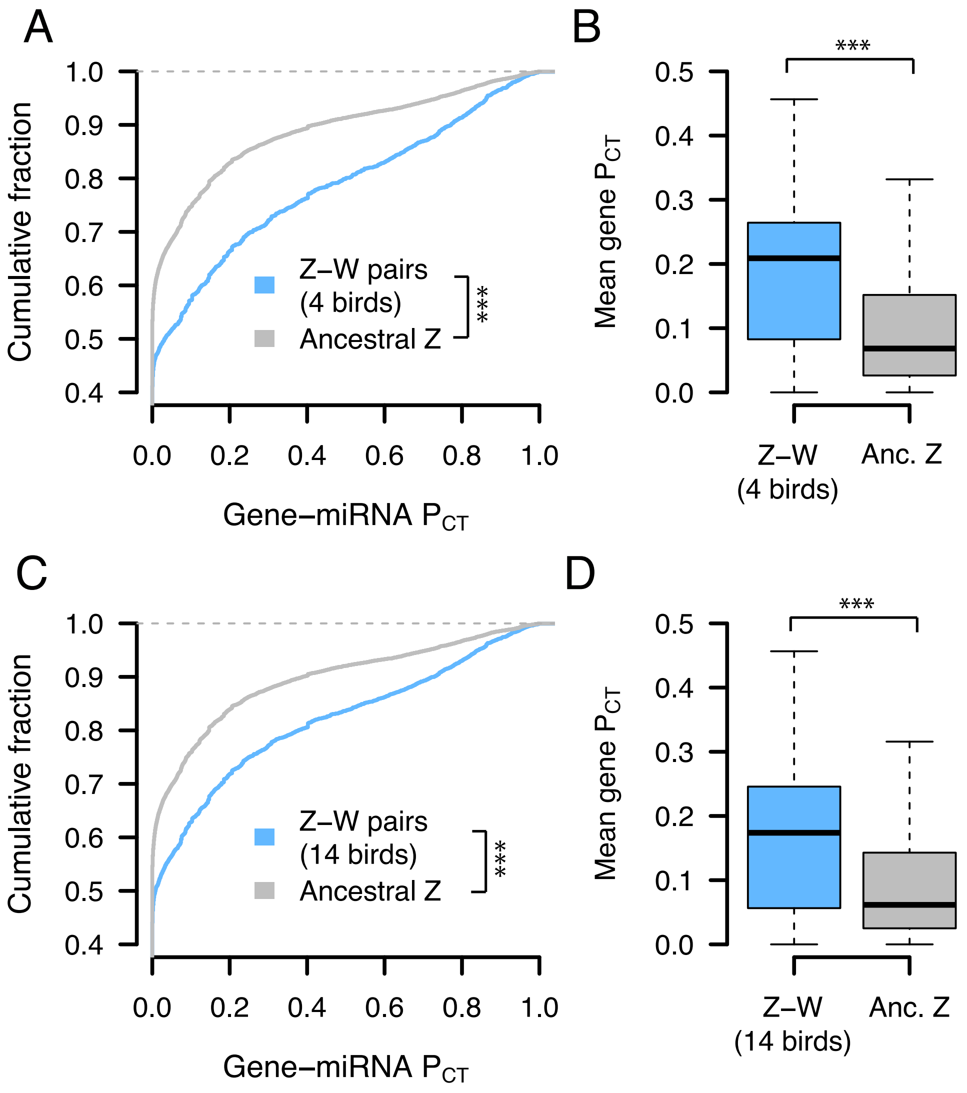

Supplemental Figure S9: P_{CT} scores of Z-W pairs across 4 and 14 birds. (A,C) P_{CT} score distributions of all gene-miRNA interactions (A) Z-W pairs including predictions from three additional birds with male and female genome sequence (n = 2,187 interactions from 78 genes) and other ancestral Z genes (n = 15,357 interactions from 607 genes), or (C) Z-W pairs including read depth-based predictions from 10 additional birds with only female genome sequence (n = 4,458 interactions from 157 genes) and other ancestral Z genes (n = 13,086 interactions from 528 genes) *** $p < 0.001$, two-sided Kolmogorov-Smirnov test. (B,D) Gene-level mean P_{CT} scores. *** $p < 0.01$, two-sided Wilcoxon rank-sum test.
