# Supplementary material for: Conserved microRNA targeting reveals preexisting gene dosage sensitivities that shaped amniote sex chromosome evolution
Source: Genome Res. 2018 Apr;28(4):474–83. doi: 10.1101/gr.230433.117 (PMC5880238; doi:10.1101/gr.230433.117)
Supplement: Supplemental Material [file supp_gr.230433.117_Supplemental_Fig_S10.pdf]

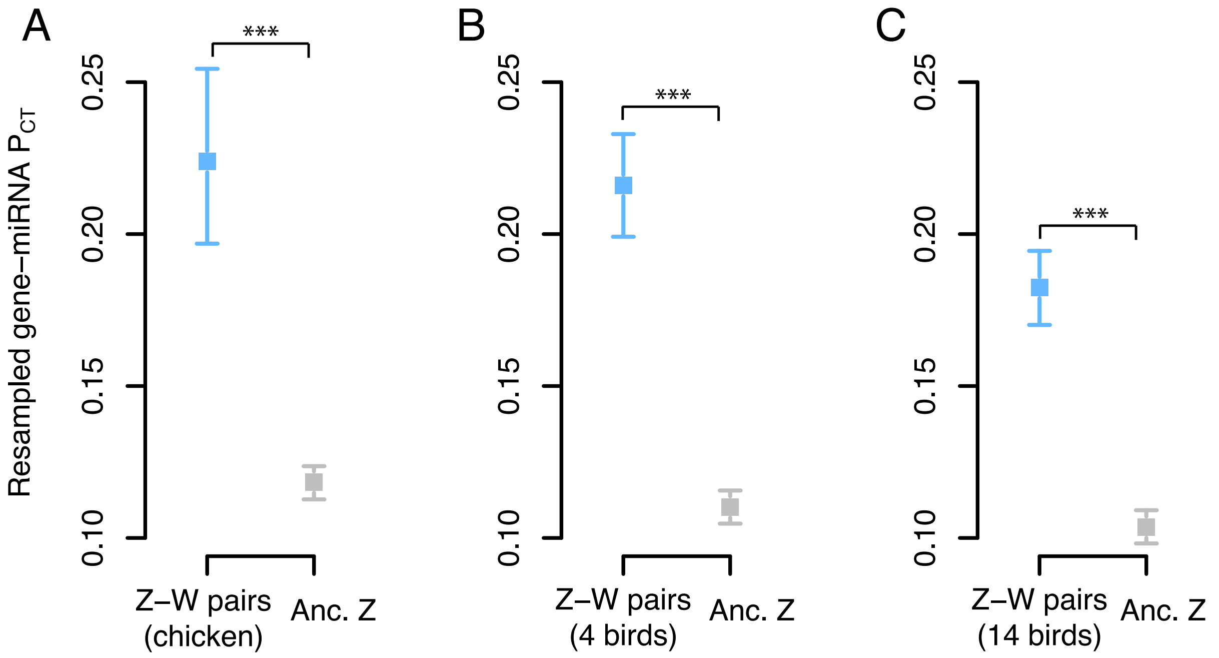

Supplemental Figure S10: Resampled mean P_{CT} scores of Z-linked genes. Gene sets: (A) chicken Z-W pairs (n = 28 genes) and other ancestral Z genes (n = 657 genes), (B) Z-W pairs across four birds (n = 78 genes) compared to the remainder of ancestral Z genes (n = 607 genes), and (C) Z-W pairs across 14 birds (n = 157 genes) compared to the remainder of ancestral Z genes (n = 528 genes). Points and error bars represent the median and 95% confidence intervals from 1,000 gene samplings with replacement. *** $p < 0.001$, empirical p-value computed as the fraction of random non-overlapping gene sets with a median difference in P_{CT} score at least as large as the true difference.
