# Supplementary material for: Conserved microRNA targeting reveals preexisting gene dosage sensitivities that shaped amniote sex chromosome evolution
Source: Genome Res. 2018 Apr;28(4):474–83. doi: 10.1101/gr.230433.117 (PMC5880238; doi:10.1101/gr.230433.117)
Supplement: Supplemental Material [file supp_gr.230433.117_Supplemental_Fig_S11.pdf]

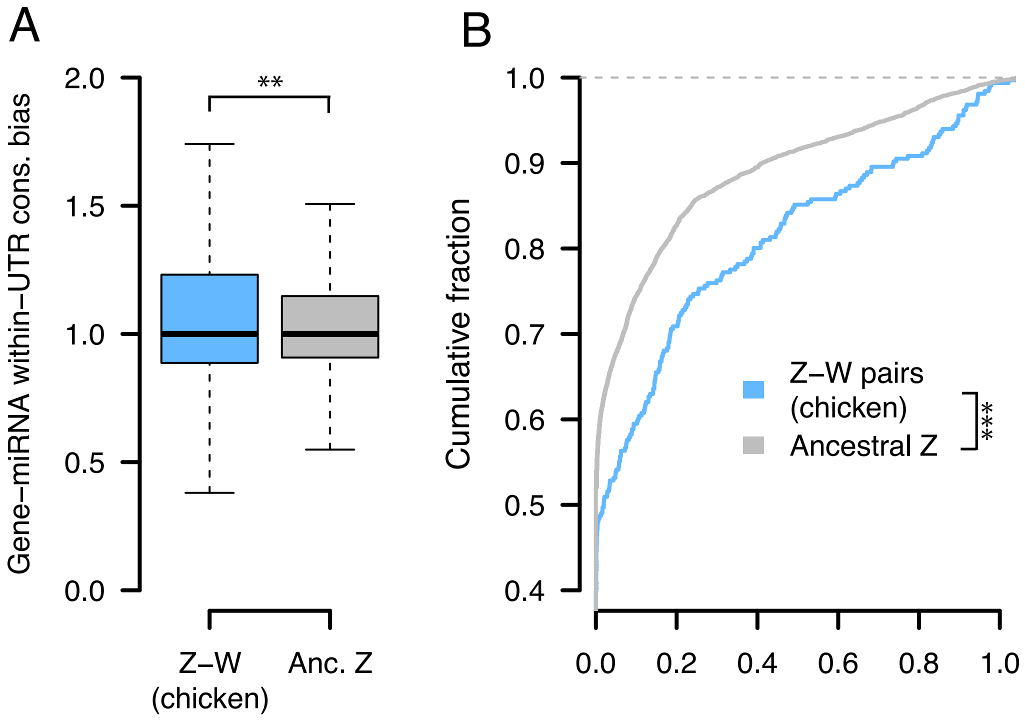

Supplemental Figure S11: Variation in within-UTR conservation cannot account for observed differences in P_{CT} score among classes of Z-linked genes. (A) Boxplots of within-UTR conservation bias (see Methods) for all gene-miRNA interactions involving chicken Z-W pairs or other ancestral X genes. Numbers of interactions and genes as in Figure 4A. ** $p < 0.01$, two-side Wilcoxon rank-sum test. (B) Comparisons of P_{CT} scores normalized by within-UTR bias. *** $p < 0.001$, two-sided Kolmogorov-Smirnov test.
