# Supplementary material for: Conserved microRNA targeting reveals preexisting gene dosage sensitivities that shaped amniote sex chromosome evolution
Source: Genome Res. 2018 Apr;28(4):474–83. doi: 10.1101/gr.230433.117 (PMC5880238; doi:10.1101/gr.230433.117)
Supplement: Supplemental Material [file supp_gr.230433.117_Supplemental_Fig_S12.pdf]

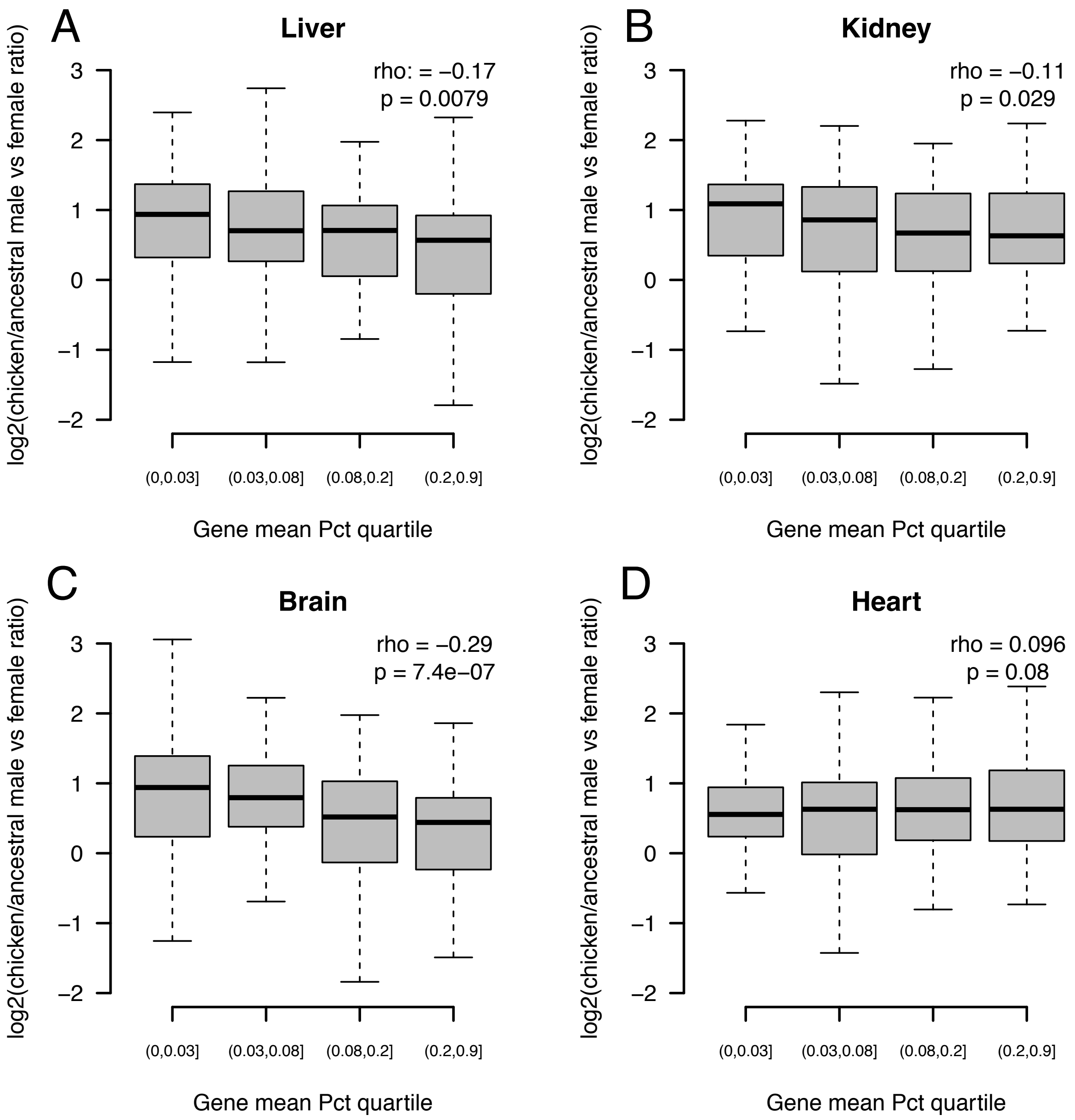

Supplemental Figure S12: Correlation of Z-linked gene-specific dosage compensation with gene-level P_{CT} score. Distributions of chicken male/female expression ratio, normalized to that of human and anolis (y-axis) as a function of mean gene-level P_{CT} quartile (x-axis) for all expressed Z-linked gene with no W homolog. Expression ratios are plotted on a $\log_2$ scale; values closer to 0 imply more effective dosage compensation following W gene loss.
