# Supplementary material for: Conserved microRNA targeting reveals preexisting gene dosage sensitivities that shaped amniote sex chromosome evolution
Source: Genome Res. 2018 Apr;28(4):474–83. doi: 10.1101/gr.230433.117 (PMC5880238; doi:10.1101/gr.230433.117)
Supplement: Supplemental Material [file supp_gr.230433.117_Supplemental_Fig_S13.pdf]

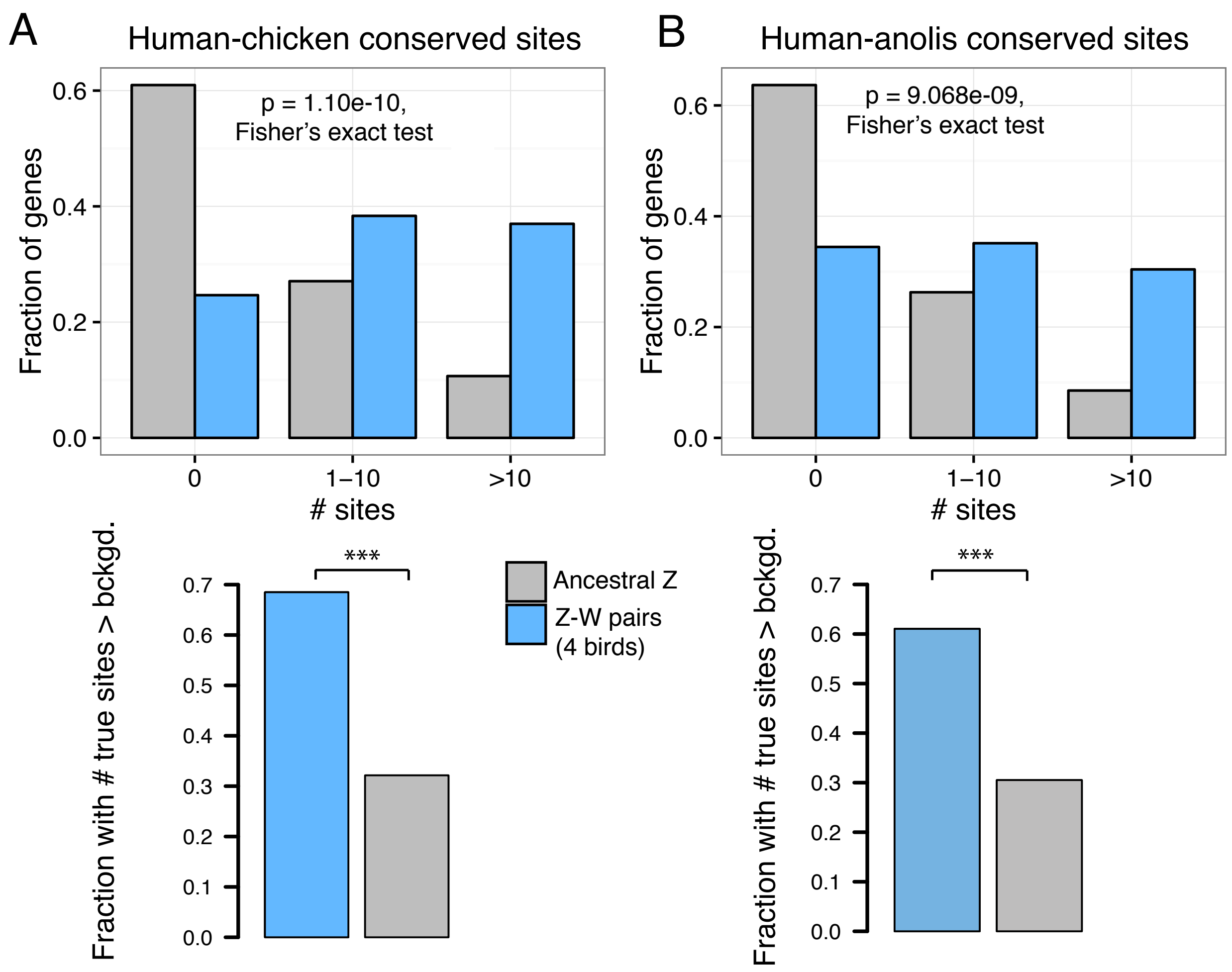

Supplemental Figure S13: Ancestral miRNA targeting of Z-W pairs across 4 birds. (A) Distributions of sites conserved between 3` UTRs of human and chicken orthologs (top) or comparisons to background expectation (bottom, see Methods) for Z-W pairs across chicken and three additional birds with male and female genome sequence (4 birds, n = 73) and other ancestral Z genes (n = 532). (D) Statistics as in (C), but using sites conserved between human and anolis 3` UTRs; Z-W pairs across 4 birds (n = 73), other ancestral Z genes (n = 527). *** $p < 0.001$, two-sided Fisher's exact test.
