# Supplementary material for: Conserved microRNA targeting reveals preexisting gene dosage sensitivities that shaped amniote sex chromosome evolution
Source: Genome Res. 2018 Apr;28(4):474–83. doi: 10.1101/gr.230433.117 (PMC5880238; doi:10.1101/gr.230433.117)
Supplement: Supplemental Material [file supp_gr.230433.117_Supplemental_Fig_S14.pdf]

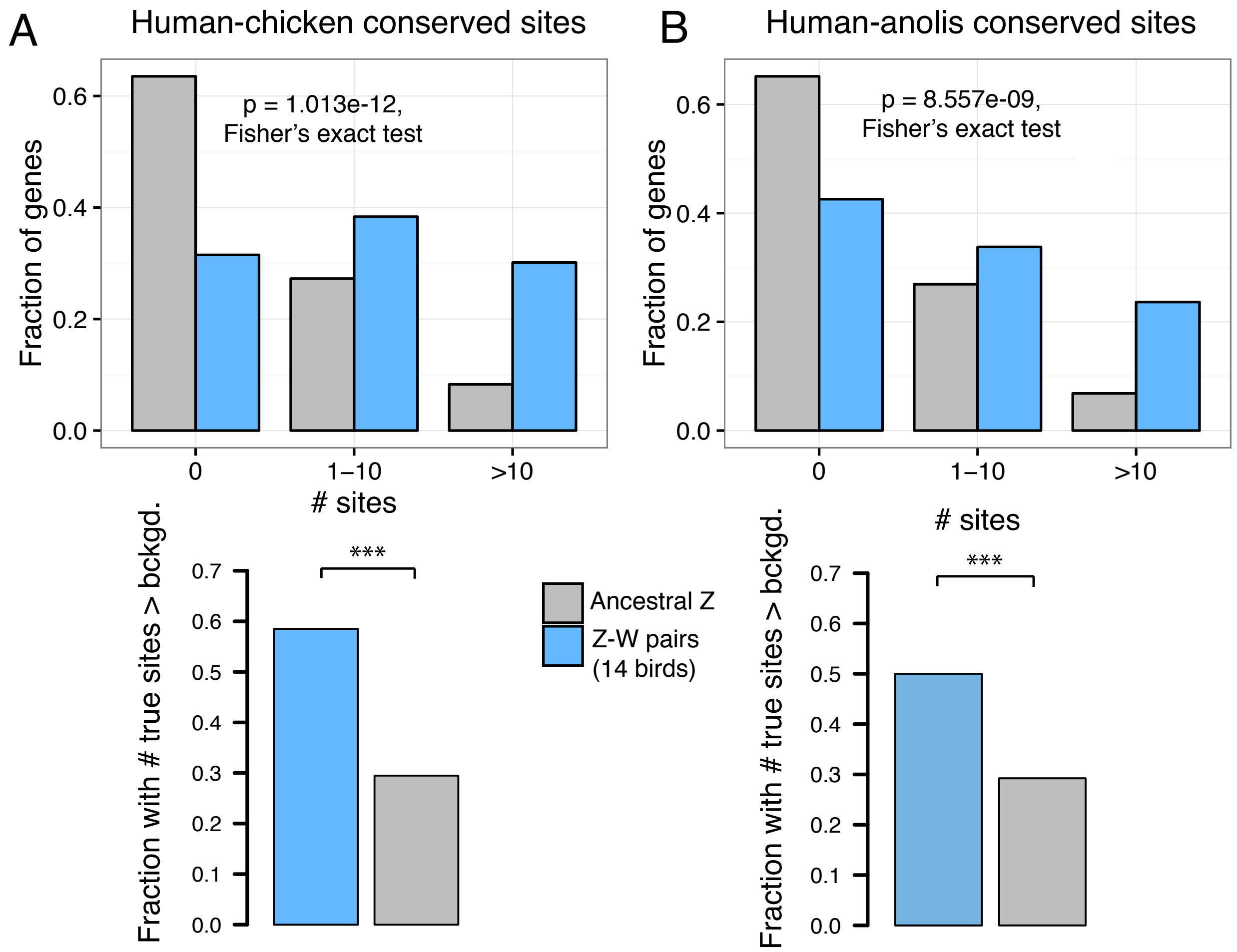

Supplemental Figure S14: Ancestral miRNA targeting of predicted Z-W pairs across 14 birds. (A) Distributions of sites conserved between 3` UTRs of human and chicken orthologs (top) or comparisons to background expectation (bottom, see Methods) for Z-W pairs in chicken, predicted in three additional birds with male and female genome sequence, and predicted based on read depth from 10 additional birds with only female genome sequence (14 birds, n = 147) and other ancestral Z genes (n = 458). (D) Statistics as in (C), but using sites conserved between human and anolis 3` UTRs; Z-W pairs across 14 birds (n = 147), other ancestral Z genes (n = 453).
