# Supplementary material for: Conserved microRNA targeting reveals preexisting gene dosage sensitivities that shaped amniote sex chromosome evolution
Source: Genome Res. 2018 Apr;28(4):474–83. doi: 10.1101/gr.230433.117 (PMC5880238; doi:10.1101/gr.230433.117)
Supplement: Supplemental Material [file supp_gr.230433.117_Supplemental_Fig_S15.pdf]

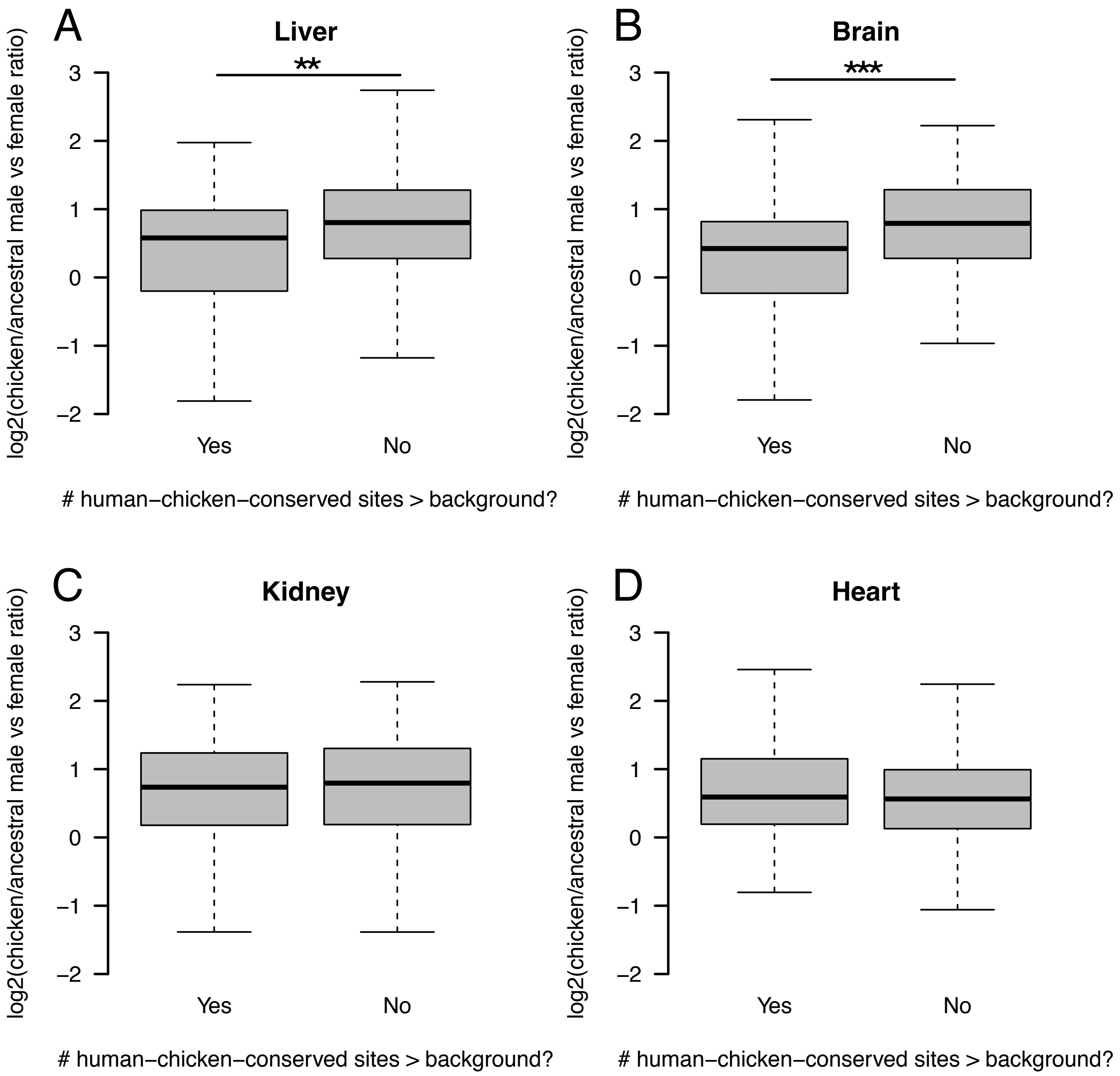

Supplemental Figure S15: Correlation of Z-linked gene-specific dosage compensation with human-chicken-conserved site excess. Distributions of chicken male/female expression ratios, normalized to those of human and anolis (y-axis) for expressed Z-linked genes with no W homolog with (left) or without (right) an excess of human-chicken-conserved miRNA sites. ** $p < 0.01$, *** $p < 0.0001$, Wilcoxon rank-sum test.
