# Supplementary material for: Conserved microRNA targeting reveals preexisting gene dosage sensitivities that shaped amniote sex chromosome evolution
Source: Genome Res. 2018 Apr;28(4):474–83. doi: 10.1101/gr.230433.117 (PMC5880238; doi:10.1101/gr.230433.117)
Supplement: Supplemental Material [file supp_gr.230433.117_Supplemental_Fig_S18.pdf]

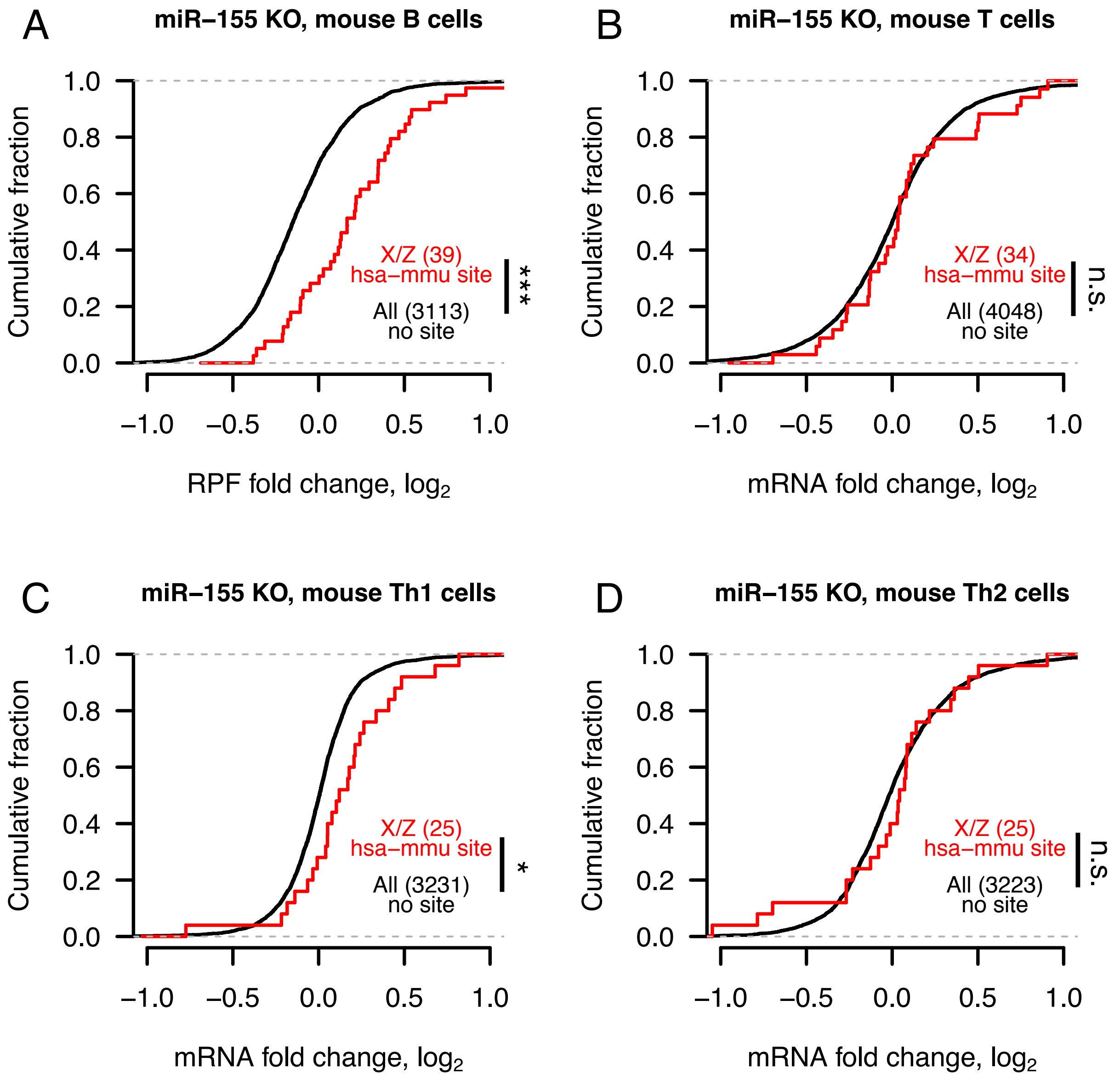

Supplemental Figure S18: Changes in mRNA stability and translational efficiency and gene expression following miR-155 knockout in mouse immune cells. In each case, mouse orthologs of X- or Z-linked genes containing a human-mouse-conserved (hsa-mmu) miR-155 site were compared to mouse genes containing only nonconserved miR-155 sites. * $p < 0.05$, *** $p < 0.001$, two-sided Kolmogorov-Smirnov test.
