# Supplementary material for: Conserved microRNA targeting reveals preexisting gene dosage sensitivities that shaped amniote sex chromosome evolution
Source: Genome Res. 2018 Apr;28(4):474–83. doi: 10.1101/gr.230433.117 (PMC5880238; doi:10.1101/gr.230433.117)
Supplement: Supplemental Material [file supp_gr.230433.117_Supplemental_Fig_S19.pdf]

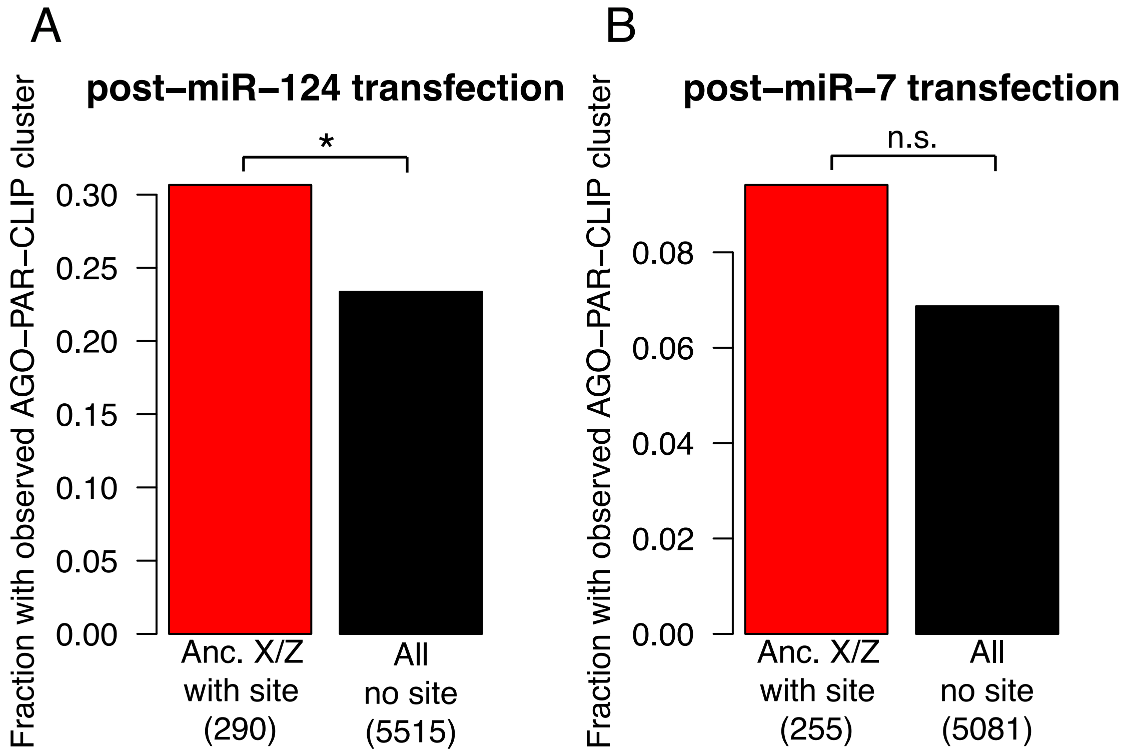

Supplemental Figure S19: Argonaute binding measured by high-throughput crosslinking-immunoprecipitation (CLIP) following miRNA transfection in HEK293 cells. * $p < 0.05$, two-sided Fisher's exact test.
