# Supplementary material for: Conserved microRNA targeting reveals preexisting gene dosage sensitivities that shaped amniote sex chromosome evolution
Source: Genome Res. 2018 Apr;28(4):474–83. doi: 10.1101/gr.230433.117 (PMC5880238; doi:10.1101/gr.230433.117)
Supplement: Supplemental Material [file supp_gr.230433.117_Supplemental_Methods.docx]

**Human genic copy number variation**

We downloaded Exome Aggregation Consortium (ExAC) data from (ftp://ftp.broadinstitute.org/pub/ExAC_release/release0.3.1/cnv/). We used the publicly available genic deletion counts but re-computed genic duplication counts using only full duplications, reasoning that partial duplications are unlikely to result in increased dosage of the full gene product. We thus required that an individual duplication fully overlapped the longest protein-coding transcript (GENCODE v19) of a gene using BEDtools (RRID:SCR_006646) (Quinlan & Hall, 2010). We removed genes flagged by ExAC as lying in known regions of recurrent CNVs. This yielded 4,118 genes within duplications and deletions, 3,976 genes within deletions but not duplications, 2,916 genes within duplications but not deletions, and 3,510 genes not subject to duplication or deletion.

**X-linked gene sets**

Analyses of conserved miRNA targeting based on multiple species alignments are unreliable for multicopy or ampliconic genes due to ambiguous sequence alignment between species. To avoid such issues, we first removed multicopy and ampliconic genes (Mueller et al., 2013) from a previously published set of human X genes present in the amniote ancestor (Bellott et al., 2014). We then excluded genes in the human pseudoautosomal (PAR) regions since these genes have not been exposed to the same evolutionary forces as genes in regions where X-Y recombination has been suppressed. Of the remaining ancestral X genes, we classified the 15 genes with human Y-linked homologs as X-Y pairs. We also analyzed the larger set of 32 X-Y pairs across eight mammals (human, chimpanzee, rhesus macaque, marmoset, mouse, rat, bull, and opossum) with sequenced Y Chromosomes (Bellott et al., 2014).

To classify ancestral X-linked genes without Y homologs as subject to or escaping XCI in humans, we used a collection of consensus XCI calls which aggregate the results of three studies (Carrel & Willard, 2005; Cotton et al., 2013, 2015) assaying XCI escape (Balaton, Cotton, & Brown, 2015). Out of 472 ancestral X genes without a human Y homolog assigned an XCI status by Balaton et al. (Balaton et al., 2015), 329 were subject to XCI (“Subject” or “Mostly subject” in Balaton et al.), 26 displayed variable escape (“Variable escape” or “Mostly variable escape”) from XCI, and 30 showed consistent escape (“Escape” or “Mostly escape”). We excluded 40 ancestral X genes with a “Discordant” XCI status as assigned by Balaton et al. In the main text, we present results obtained after combining both variable and consistent escape calls from Balaton et al. into one class, yielding the following counts: 15 X-Y pairs, 329 ancestral X genes subject to XCI, and 56 ancestral X genes with evidence of escape from XCI. We also performed analyses considering escape and variable escape genes separately.

**Z-linked gene sets**

We previously refined the ancestral gene content of the avian sex chromosomes to 685 Z-linked genes with human orthologs by sequencing of the chicken Z Chromosome and analysis of 13 other avian species with published female genomes (Bellott et al., 2017). Of these 685 ancestral Z genes, 28 retained a homolog on the fully sequenced chicken W Chromosome. Including three additional avian species in which candidate W-linked genes were ascertained by directly comparing male and female genome assemblies results in a total of 78 W-linked genes. Including another 10 avian species in which W-linkage was inferred by read depth changes in a female genome results in a total of 157 W-linked genes.

**microRNA target site P_CT_ scores**

Pre-calculated P_CT_ scores for all gene-miRNA family interactions were obtained from TargetScanHuman v7.1 (RRID:SCR_010845) (<http://www.targetscan.org/vert_71/vert_71_data_download/Summary_Counts.all_predictions.txt.zip>), (Friedman, Farh, Burge, & Bartel, 2009). We excluded mammalian-specific miRNA families based on classifications by Friedman et al (Friedman et al., 2009) and updated in TargetScanHuman v7.1(Agarwal, Bell, Nam, & Bartel, 2015). To account for gene-specific variability in the number and P_CT_ score of gene-miRNA interactions within a group of genes, we sampled 1000x with replacement from the same group of genes and computed the mean gene-miRNA P_CT_ score for all associated gene miRNA interactions from each sampling. These 1000 samplings were then used to estimate the median resampled gene-miRNA P_CT_ and 95% confidence intervals.

**Human-chicken conserved microRNA target sites**

Site-wise alignment information was obtained from TargetScanHuman v7.1 (http://www.targetscan.org/vert_71/vert_71_data_download/Conserved_Family_Info.txt.zip). To determine which target sites are present in the 3` UTRs of both human and chicken orthologs, we counted, for genes with both a human and chicken ortholog, the number of miRNA interactions that had at least one target site in both human and chicken. To control for gene-specific background 3` UTR conservation, we generated six control k-mers for each miRNA family seed sequence that were matched exactly for nucleotide and CpG content. Six was the maximum number of unique control k-mers that could be generated for all sequences. We repeated the above counting analysis with each of the control k-mers using scripts from TargetScan, and compared, for each gene, the observed number of human-chicken-conserved miRNA interactions (the observed conservation signal) to the average number from controls (the background conservation). This same procedure was repeated for alternative pairs of species considered (opossum-chicken and human-anolis lizard).

**Human-chicken conserved microRNA target sites**

Site-wise alignment information was obtained from TargetScanHuman v7.1 (http://www.targetscan.org/vert_71/vert_71_data_download/Conserved_Family_Info.txt.zip). To determine which target sites are present in the 3` UTRs of both human and chicken orthologs, we counted, for genes with both a human and chicken ortholog, the number of miRNA interactions that had at least one target site in both human and chicken. To control for gene-specific background 3` UTR conservation, we generated six control k-mers for each miRNA family seed sequence that were matched exactly for nucleotide and CpG content. Six was the maximum number of unique control k-mers that could be generated for all sequences. We repeated the above counting analysis with each of the control k-mers using scripts from TargetScan, and compared, for each gene, the observed number of human-chicken-conserved miRNA interactions (the observed conservation signal) to the average number from controls (the background conservation). This same procedure was repeated for alternative pairs of species considered (opossum-chicken and human-anolis lizard).

**Variation in within-UTR conservation bias**

The P_CT_ of a given miRNA target site depends on the conservation of the site, as measured by the total branch length of the phylogenetic tree containing the target sites (branch length score, BLS) relative to the mean BLS of the whole 3` UTR. To address the possibility that non-uniformity in the regional BLS could artificially inflate or deflate conservation scores of certain target sites, we implemented a step-detection algorithm to segment 3` UTRs into regions of homogeneous BLS values. In order to call steps within a 3` UTR, we computed the t-test p-value between the BLS values of the 50-nt window upstream and downstream of each nucleotide position in the 3` UTR. Transitions were called at a log p-value cutoff of -15. Because of noise in the BLS signal, the log p-value often dips below -15 several times around each transition. If more than 1 position met the cutoff within 100 nucleotides of each other, we took only the one with the smaller p-value. We then computed, for each miRNA site, the ratio of the mean BLS of its section to that of the entire 3` UTR; we term this statistic the “within-UTR conservation bias”. Values of this statistic greater than 1 indicate that the P_CT_ overestimates the relative conservation of a given target site, while values less than 1 indicate that the P_CT_ underestimates conservation. For gene-miRNA interactions with multiple sites, we used the mean within-UTR conservation bias for all sites. We also repeated P_CT_ score comparisons between classes of X- and Z-linked genes with P_CT_ scores normalized by the corresponding gene-miRNA within-UTR conservation bias (Supplemental Figure S6C, Supplemental Figure S9B).

**Gene expression profiling and crosslinking datasets**

Fold-changes in mRNA expression from a compendium of small RNA (sRNA) transfections (corresponding to twelve different miRNAs) in HeLa cells were obtained from Agarwal and colleagues (Agarwal et al., 2015) (GSM210904, GSM37601, GSM210913, GSM210903, GSM210911, GSM210898, GSM210897, GSM210897, GSM210901, GSM210909, GSM119747; E-MEXP-1402(1595297513)). Further datasets describing the effects of transfecting miR-103 in HCT116 cells (Linsley et al., 2007) (GSM156580), knocking down miR-92a in HEK293 cells (Hafner et al., 2010) ([GSM538818](http://www.ncbi.nlm.nih.gov/geo/query/acc.cgi?acc=GSM538818)), transfecting miR-7 or miR-124 in HEK293 cells (Hausser, Landthaler, Jaskiewicz, Gaidatzis, & Zavolan, 2009) ([GSM363763](http://www.ncbi.nlm.nih.gov/geo/query/acc.cgi?acc=GSM363763),

[GSM363766](http://www.ncbi.nlm.nih.gov/geo/query/acc.cgi?acc=GSM363766), [GSM363769](http://www.ncbi.nlm.nih.gov/geo/query/acc.cgi?acc=GSM363769), [GSM363772](http://www.ncbi.nlm.nih.gov/geo/query/acc.cgi?acc=GSM363772), [GSM363775](http://www.ncbi.nlm.nih.gov/geo/query/acc.cgi?acc=GSM363775), [GSM363778](http://www.ncbi.nlm.nih.gov/geo/query/acc.cgi?acc=GSM363778)), or of knocking out miR-155 in mouse B cells (Eichhorn et al., 2014) ([GSM1479572](http://www.ncbi.nlm.nih.gov/geo/query/acc.cgi?acc=GSM1479572), [GSM1479576](http://www.ncbi.nlm.nih.gov/geo/query/acc.cgi?acc=GSM1479576), [GSM1479580](http://www.ncbi.nlm.nih.gov/geo/query/acc.cgi?acc=GSM1479580), [GSM1479584](http://www.ncbi.nlm.nih.gov/geo/query/acc.cgi?acc=GSM1479584)), T cells (Loeb et al., 2012) ([GSM1012118](http://www.ncbi.nlm.nih.gov/geo/query/acc.cgi?acc=GSM1012118), [GSM1012119](http://www.ncbi.nlm.nih.gov/geo/query/acc.cgi?acc=GSM1012119), [GSM1012120](http://www.ncbi.nlm.nih.gov/geo/query/acc.cgi?acc=GSM1012120), [GSM1012121](http://www.ncbi.nlm.nih.gov/geo/query/acc.cgi?acc=GSM1012121), [GSM1012122](http://www.ncbi.nlm.nih.gov/geo/query/acc.cgi?acc=GSM1012122), [GSM1012123](http://www.ncbi.nlm.nih.gov/geo/query/acc.cgi?acc=GSM1012123)), or Th1 and Th2 cells (Rodriguez et al., 2007) (E-TABM-232), processed as described in (Agarwal et al., 2015), were provided by V. Agarwal. Targets for the PAR-CLIP study (Hafner et al., 2010) were inferred from an online resource of HEK293 clusters observed after transfection of either miR-124 ([http://www.mirz.unibas.ch/restricted/clipdata/RESULTS/miR124_TRANSFECTION/miR124_TRANSFECTION.html](http://www.mirz.unibas.ch/restricted/clipdata/RESULTS/miR124_TRANSFECTION/miR124_TRANSFECTION.html)) or miR-7 ([http://www.mirz.unibas.ch/restricted/clipdata/RESULTS/miR7_TRANSFECTION/miR7_TRANSFECTION.html](http://www.mirz.unibas.ch/restricted/clipdata/RESULTS/miR7_TRANSFECTION/miR7_TRANSFECTION.html)).

**Assessing Z-linked dosage compensation using cross-species RNA-sequencing data**

Raw RNA-seq reads of male and female samples from 4 somatic tissues (liver, brain, kidney, and heart) from human, chicken and anolis were obtained from (Marin et al., 2017) (GSE97367). Kallisto was used to pseudomap reads and quantify transcript abundances (human, GENCODE v26; chicken and anolis, Ensembl 87), with the following options: “--bias”, “--single”, “-l 200”, “-s 20.” Transcripts were summed to the gene-level using the tximport R package with the option “lengthscaledTPM.” Ensembl one-to-one orthologs were used, except for ancestral Z-linked genes, where orthology assignments in Supplemental Table S4 were used. Within each tissue, gene-level counts were normalized across species using the trimmed median of means (TMM) method in the edgeR R package. Genes were only considered for analysis if they were expressed > 1 TPM in all human, chicken, and anolis samples from that tissue. The limma/voom R package was used to quantify the male/female expression ratio in chicken relative to the male/female ratio in human and anolis.

Agarwal, V., Bell, G. W., Nam, J., and Bartel, D. P. (2015). Predicting effective microRNA target sites in mammalian mRNAs. *eLife*, *4*, e05005. https://doi.org/10.7554/eLife.05005

Balaton, B. P., Cotton, A. M., and Brown, C. J. (2015). Derivation of consensus inactivation status for X-linked genes from genome-wide studies. *Biology of Sex Differences*, *6*, 35. https://doi.org/10.1186/s13293-015-0053-7

Bellott, D. W., Hughes, J. F., Skaletsky, H., Brown, L. G., Pyntikova, T., Cho, T.-J., Koutseva, N., Zaghlul, S., Graves, T., Rock, S., Kremitzki, C., Fulton, R. S., Dugan, S., Ding, Y., Morton, D., Khan, Z., Lewis, L., … Page, D. C. (2014). Mammalian Y chromosomes retain widely expressed dosage-sensitive regulators. *Nature*, *508*(7497), 494–499. https://doi.org/10.1038/nature13206

Bellott, D. W., Skaletsky, H., Cho, T.-J., Brown, L., Locke, D., Chen, N., Galkina, S., Pyntikova, T., Koutseva, N., Graves, T., Kremitzki, C., Warren, W. C., Clark, A. G., Gaginskaya, E., Wilson, R. K., and Page, D. C. (2017). Avian W and mammalian Y chromosomes convergently retained dosage-sensitive regulators. *Nature Genetics*, *in press*.

Carrel, L., and Willard, H. F. (2005). X-inactivation profile reveals extensive variability in X-linked gene expression in females. *Nature*, *434*(March), 400–404. https://doi.org/10.1038/nature03479

Cotton, A. M., Ge, B., Light, N., Adoue, V., Pastinen, T., and Brown, C. J. (2013). Analysis of expressed SNPs identifies variable extents of expression from the human inactive X chromosome. *Genome Biology*, *14*(11), R122. https://doi.org/10.1186/gb-2013-14-11-r122

Cotton, A. M., Price, E. M., Jones, M. J., Balaton, B. P., Kobor, M. S., and Brown, C. J. (2015). Landscape of DNA methylation on the X chromosome reflects CpG density, functional chromatin state and X-chromosome inactivation. *Human Molecular Genetics*, *24*(6), 1528–1539. https://doi.org/10.1093/hmg/ddu564

Eichhorn, S. W., Guo, H., McGeary, S. E., Rodriguez-Mias, R. a, Shin, C., Baek, D., Hsu, S.-H., Ghoshal, K., Villén, J., and Bartel, D. P. (2014). mRNA Destabilization Is the Dominant Effect of Mammalian MicroRNAs by the Time Substantial Repression Ensues. *Molecular Cell*. https://doi.org/10.1016/j.molcel.2014.08.028

Friedman, R. C., Farh, K. K.-H., Burge, C. B., and Bartel, D. P. (2009). Most mammalian mRNAs are conserved targets of microRNAs. *Genome Research*, *19*(1), 92–105. https://doi.org/10.1101/gr.082701.108

Hafner, M., Landthaler, M., Burger, L., Khorshid, M., Berninger, P., Rothballer, A., Jr, M. A., Munschauer, M., Ulrich, A., Wardle, G. S., Dewell, S., Zavolan, M., and Tuschl, T. (2010). Transcriptome-wide identification of RNA-binding protein and microRNA target sites by PAR-CLIP. *Cell*, *141*(1), 129–141. https://doi.org/10.1016/j.cell.2010.03.009.Transcriptome-wide

Hausser, J., Landthaler, M., Jaskiewicz, L., Gaidatzis, D., and Zavolan, M. (2009). mRNA binding of Argonaute / EIF2C − miRNA complexes and the degradation of miRNA targets Relative contribution of sequence and structure features to the mRNA binding of Argonaute / EIF2C – miRNA complexes and the degradation of miRNA targets. *Genome Research*, 2009–2020. https://doi.org/10.1101/gr.091181.109

Linsley, P. S., Schelter, J., Burchard, J., Kibukawa, M., Martin, M. M., Bartz, S. R., Johnson, J. M., Cummins, J. M., Raymond, C. K., Dai, H., Chau, N., Cleary, M., Jackson, A. L., Carleton, M., and Lim, L. (2007). Transcripts targeted by the microRNA-16 family cooperatively regulate cell cycle progression. *Mol Cell Biol*, *27*(6), 2240–2252. https://doi.org/10.1128/MCB.02005-06

Loeb, G. B., Khan, A. A., Canner, D., Hiatt, J. B., Shendure, J., Darnell, R. B., Leslie, C. S., and Rudensky, A. Y. (2012). Transcriptome-wide miR-155 Binding Map Reveals Widespread Noncanonical MicroRNA Targeting. *Molecular Cell*, *48*(5), 760–770. https://doi.org/http://dx.doi.org/10.1016/j.molcel.2012.10.002

Marin, R., Cortez, D., Lamanna, F., Pradeepa, M. M., Leushkin, E., Julien, P., Liechti, A., Halbert, J., Brüning, T., Mössinger, K., Trefzer, T., Conrad, C., Kerver, H. N., Wade, J., Tschopp, P., and Kaessmann, H. (2017). Convergent origination of a Drosophila -like dosage compensation mechanism in a reptile lineage. *Genome Research*, 1–14. https://doi.org/10.1101/gr.223727.117

Mueller, J. L., Skaletsky, H., Brown, L. G., Zaghlul, S., Rock, S., Graves, T., Auger, K., Warren, W. C., Wilson, R. K., and Page, D. C. (2013). Independent specialization of the human and mouse X chromosomes for the male germ line. *Nature Genetics*, *45*(9), 1083–1087. https://doi.org/10.1038/ng.2705

Quinlan, A. R., and Hall, I. M. (2010). BEDTools: A flexible suite of utilities for comparing genomic features. *Bioinformatics*, *26*(6), 841–842. https://doi.org/10.1093/bioinformatics/btq033

Rodriguez, A., Vigorito, E., Clare, S., Warren, M. V, Couttet, P., Soond, D. R., Dongen, S. Van, Grocock, R. J., Das, P. P., Miska, E. A., Vetrie, D., Okkenhaug, K., Enright, A. J., Dougan, G., Turner, M., and Bradley, A. (2007). Requirement of bic/microRNA-155 for normal immune function. *Science*, *981*(April), 608–611. https://doi.org/10.1126/science.1139253
